# The role of Golgi complex proteins in cell division and consequences of their dysregulation

**DOI:** 10.3389/fcell.2024.1513472

**Published:** 2025-01-07

**Authors:** Roberta Iannitti, Fabiola Mascanzoni, Antonino Colanzi, Daniela Spano

**Affiliations:** Department of Biomedical Sciences (DSB), Institute of Experimental Endocrinology and Oncology “G. Salvatore” (IEOS), National Research Council (CNR), Naples, Italy

**Keywords:** Golgi complex, mitosis, meiosis, cell fate, cancer

## Abstract

The GC (Golgi complex) plays a pivotal role in the trafficking and sorting of proteins and lipids until they reach their final destination. Additionally, the GC acts as a signalling hub to regulate a multitude of cellular processes, including cell polarity, motility, apoptosis, DNA repair and cell division. In light of these crucial roles, the GC has garnered increasing attention, particularly given the evidence that a dysregulation of GC-regulated signalling pathways may contribute to the onset of various pathological conditions. This review examines the functions of the GC and GC-localised proteins in regulating cell cycle progression, in both mitosis and meiosis. It reviews the involvement of GC-resident proteins in the formation and orientation of the spindle during cell division. In light of the roles played by the GC in controlling cell division, this review also addresses the involvement of the GC in cancer development. Furthermore, TCGA (The Cancer Genome Atlas) database has been queried in order to retrieve information on the genetic alterations and the correlation between the expression of GC-localised proteins and the survival of cancer patients. The data presented in this review highlight the relevance of the GC in regulating cell cycle progression, cellular differentiation and tumourigenesis.

## 1 Introduction

In vertebrate cells, the GC (Golgi complex) is composed of stacks of flattened cisternae that are laterally connected by tubules (Rambourg and Clermont, 1990) to form the so-called “Golgi ribbon”, which is localised near the CE (centrosome) and the nucleus (Rios, 2014). The correct architecture and positioning of the GC are essential for its functional activities and are regulated by the Golgi matrix proteins, including GRASPs (Golgi Reassembly And Stacking Proteins) and Golgins (Li et al., 2019), and MTs (microtubules) (de Forges et al., 2012; Gurel et al., 2014). MTs function is essential for maintaining the juxta-nuclear localisation of the GC. Indeed, the use of MT poisons has been shown to result in the dispersion of the GC into mini-stacks (de Forges et al., 2012; Gurel et al., 2014).

**FIGURE 1:**
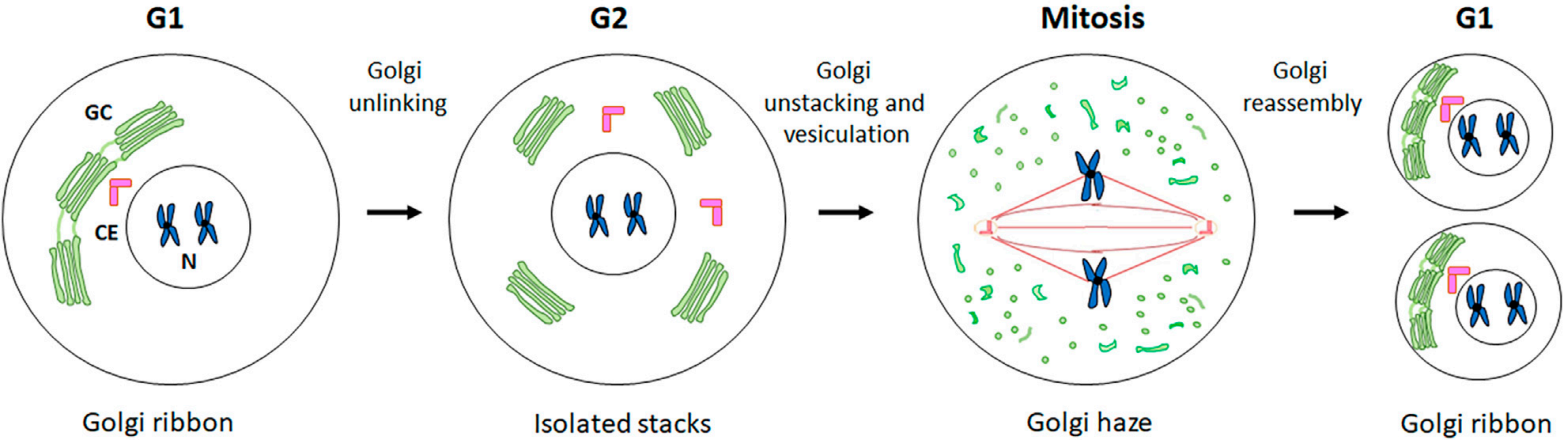
GC changes during cell cycle progression. A schematic representation of the structural and cellular localisation changes of GC during cell cycle is shown. See the text for the details.

During the different phases of the cell cycle, the GC undergoes significant structural and cellular localisation changes (Figure 1) (Li et al., 2019). During G1 phase, the GC is compact and localised in close proximity to the CE. During S phase, the GC dissociates from the CE and surrounds the nucleus. Subsequently, during G2 the GC undergoes fragmentation into isolated stacks (a process known as “Golgi unlinking”) (Ayala and Colanzi, 2017), which during mitosis are further disassembled and dispersed in the cytoplasm until the formation of the so-called “Golgi haze”. During this extensive disassembly the close interconnection between the GC, the CE and the MTs undergoes significant alterations. At the end of mitosis the GC is reassembled to form a new ribbon in the daughter cells (Nakamura et al., 2012). It seems reasonable to speculate that the close connection among the GC, the CEs and MTs is strongly linked to the inheritance of the GC during the cell cycle. Indeed, the CEs and MTs are responsible for the formation of the spindle which, beyond its well-known function in promoting the proper segregation of chromosomes during mitosis, is an indispensable track system for the correct inheritance of GC proteins involved in ribbon reassembling in the daughter cells at telophase (Mascanzoni et al., 2019).

The GC is a cellular organelle that plays a crucial role in the trafficking, sorting, modification, and targeting of lipids and proteins (De Matteis and Luini, 2008). In addition to these classical functions, there is an increasing body of evidence to suggest that the GC is involved in regulating multiple cellular processes, including cell polarity (Ravichandran et al., 2020), migration (Bui et al., 2021; Xu and Wu, 2023), autophagy (Deng et al., 2020; Lu et al., 2021), cellular wound repair and regeneration (Wijaya and Xu, 2024), apoptosis (He et al., 2020), as well as mitosis and cell growth (Ayala and Colanzi, 2017; Ayala et al., 2020; Mascanzoni et al., 2022). It is therefore evident that the GC is recognised as an active signalling hub whose dysregulation may contribute to the development of several pathological conditions, including cancer, cardiovascular illnesses and neurodegenerative diseases (Lu et al., 2018; Donizy and Marczuk, 2019; Martínez-Menárguez et al., 2019; Liu et al., 2021; 2024; Zhang, 2021; Spano and Colanzi, 2022; Mohan et al., 2023). Our previous research highlights the crucial function of the GC during the G2/M transition (Colanzi et al., 2007; Corda et al., 2012; Barretta et al., 2016; Mascanzoni et al., 2024). This process requires the proper fragmentation of this organelle to ensure the correct formation of the bipolar spindle and subsequent cytokinesis. Indeed, the Golgi unlinking activates a Golgi-localised Src, which in turn phosphorylates the mitotic serine/threonine kinase Aurora-A (AURKA) on Tyr148, thereby stimulating its recruitment at the CE and kinase activity, thus enabling CE maturation. Following autophosphorylation on Thr288, in conjunction with the binding to its activating partner TPX2 (Bayliss et al., 2003; Garrido and Vernos, 2016), AURKA acquires a fully active conformation and promotes the formation of a correct bipolar spindle, due to its interaction with CEP192, Plk1, TACC (Transforming Acidic Coiled-Coil containing protein), Neural precursor cell Expressed, NEDD1 (Developmentally Downregulated 1), Hice1 (Hec1-interacting and centrosome-associated 1) and MT-stabilizing proteins including p150glued, MAP9, RASSF1A (Ras association domain-containing protein 1) and WDR62 (tryptophan (W) aspartic acid (D) Repeat domain 62) (Joukov et al., 2014; Magnaghi-Jaulin et al., 2019). These events ultimately result in mitotic entry through the activation of Cdk1, a well-known regulator of mitosis (Barretta et al., 2016). In addition, it is established that several GC proteins are involved in the formation and positioning of the CE/MTOC that drives the spindle formation during cell division. Moreover, it is also well established that GC may act as a storage centre for both cell fate determinants and their interactors that, once released upon mitotic GC fragmentation, play a role in specifying cell fate. Consequently, the depletion or the dysfunction of these GC proteins has repercussions on the aforementioned processes, subsequently causing spindle defects that pertain to dimensions, angles, MT reorganisations and migration (Mascanzoni et al., 2022). In particular, the proper positioning and orientation of the spindle determines the cell division plane, which in turn controls cell fate decisions, morphogenesis and maintenance of tissue organisation (Bergstralh et al., 2017). It has been observed that alterations of spindle orientation impair a number of physiological processes, including gastrulation, neuronal differentiation, epithelial self-renewal and tissue stratification (Gong et al., 2004; Lechler and Fuchs, 2005; Fish et al., 2006; Cabernard and Doe, 2009). Furthermore, when spindle orientation is not solidly controlled, defective growth and differentiation occur and eventually lead to hyperproliferation and cancer (Ragkousi and Gibson, 2014; Asare et al., 2017).

This review examines the functions of GC-localised proteins in regulating cell cycle progression, both during mitosis and meiosis. It focuses on the involvement of GC-resident proteins in the correct formation, positioning and orientation of the spindle during cell division. Based on these functions, the role of GC-localised proteins in cancer is then discussed. Furthermore, TCGA (The Cancer Genome Atlas) database has been queried to gather data on the genetic alterations and correlation of GC proteins with overall survival in cancer patients.

## 2 Role of Golgi complex resident proteins in somatic cell division and cell cycle progression

**FIGURE 2:**
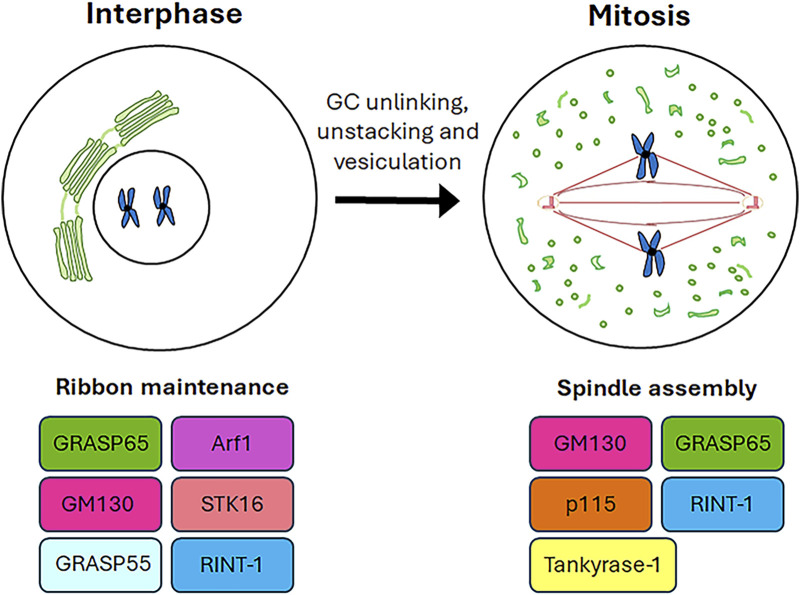
Roles of GC proteins during cell cycle. A schematic representation of the roles played by the GC-resident proteins during cell cycle progression is shown.

**FIGURE 3:**
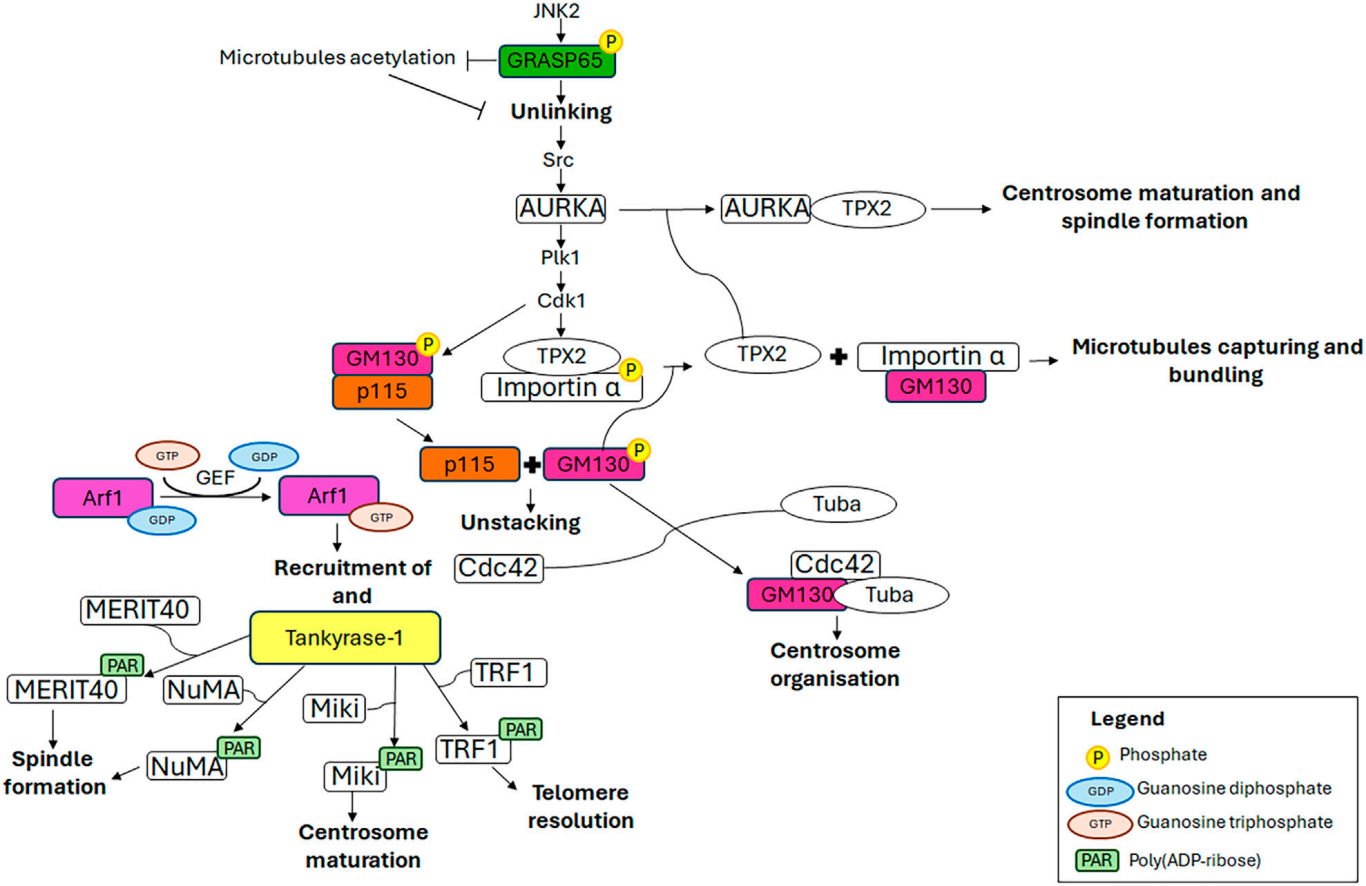
Mechanistic details of GC proteins action during mitosis. The main pathways involving the GC-resident proteins in centrosome maturation and spindle formation are displayed. Additional details on the underlying molecular mechanisms are in the text.

The available evidence increasingly points to a role for GC proteins in cell cycle progression. Indeed, the Golgins (such as GM130) and GRASP65 and GRASP55 (GRASP family of Golgi Reassembly And Stacking Proteins) play a pivotal role in maintaining the structure and dynamic nature of the GC, as well as in promoting cell cycle progression by modulating the correct formation of the spindle, chromosome segregation and cytokinesis. This section summarises the roles played by GC-resident proteins in regulating cell cycle progression as well as spindle formation during mitosis (Figure 2) and the main molecular mechanisms underlying these functions (Figure 3).

### 2.1 GRASPs (Golgi Reassembly And Stacking Proteins)

The GRASPs (Golgi Reassembly And Stacking Proteins), GRASP65 and GRASP55, are structural components of the Golgi ribbon (Barr et al., 1997; Shorter et al., 1999) localised at the *cis*-Golgi (Hu et al., 2015) and the medial/*trans*-Golgi (Zhao et al., 2017), respectively. These proteins tether adjacent membranes (Rabouille and Linstedt, 2016), thereby facilitating the formation of the Golgi ribbon. The pro-ribbon role is inhibited when these proteins are subjected to multiple phosphorylation events which lead to progressive GC disassembly during mitosis (Colanzi et al., 2007; Feinstein and Linstedt, 2008; Vinke et al., 2011; Cervigni et al., 2015; Valente and Colanzi, 2015; Ayala and Colanzi, 2017; Ayala et al., 2020). In particular, the JNK2-mediated phosphorylation of GRASP65 on Ser274 is a key event in promoting Golgi unlinking (Cervigni et al., 2015), which in turn promotes the activation and the recruitment of AURKA at the CE, as described in the introduction (Colanzi et al., 2007; Corda et al., 2012; Barretta et al., 2016; Mascanzoni et al., 2024). Consequently, AURKA depletion results in impaired GC architecture (Kimura et al., 2018), compromised CEs and spindle, and consequently, chromosome alignment defects and lagging chromosomes, ultimately leading to aneuploidy (Hoar et al., 2007). In addition, GRASP65 serves a role in the formation of the bipolar spindle and the progression of the cell cycle. Indeed, the loss of GRASP65 has been demonstrated to induce the formation of multiple aberrant spindles, which in turn causes metaphase arrest and cell death (Sütterlin et al., 2005). Furthermore, GRASP65 contributes to MTs cytoskeleton organisation, stabilising newly nucleated MTs and consequently leading to their acetylation (Ayala et al., 2019). This modification of MTs is crucial for maintaining the structure and location of the GC through the stimulation of Golgi stack clustering. During the G2 phase of the cell cycle, the acetylation of tubulin is inhibited by the JNK/ERK-mediated phosphorylation of GRASP65 on Ser274, which in turn favours the Golgi unlinking and cell cycle progression (Ayala et al., 2019). Interestingly, the stabilisation of the newly nucleated MTs is a specifically GRASP65-dependent process in which GRASP55 plays no role (Ayala et al., 2019).

Recently, a novel molecular mechanism involved in GC fragmentation through GRASP55 phosphorylation has been identified. In detail, Golgi-localised Gβγ has been demonstrated to mediate the mitotic GC fragmentation and G2/M cell cycle transition through the activation of PKD, which in turn induces the phosphorylation of GRASP55 (Rajanala et al., 2021).

### 2.2 GM130

The *cis*-Golgi-localised GM130 belongs to the Golgin family, a group of proteins that are structural components of the GC. These proteins have long coiled-coil domains that extend from the GC to connect cytoskeletal components and membranes (Lowe, 2019). GM130, comprising six coiled-coil domains, is pivotal for ribbon organisation, MTs nucleation, spindle assembly and cell polarisation, due to its interaction with a multitude of proteins, including GRASP65, Tuba, Cdc42, p115 and AKAP450 (Mascanzoni et al., 2022). Impairment of these interactions or depletion/downregulation of GM130 renders cells more susceptible to autophagy, tumour formation, metastasis (Brandstaetter et al., 2014) and altered trafficking, which causes GC disruption and subsequent neurodegeneration (Liu C. et al., 2017). For instance, it has been reported that the loss of GM130 can hamper cell polarity in some breast cancer cells, thus impacting on their cell migration and invasion, underlying that losing GM130 as a regulator of polarity renders cells more prone to accumulate defects that culminate in tumourigenesis and metastasis (Baschieri et al., 2015). Moreover, it has been observed that the loss of GM130 in a knockout mouse model impairs GC structure of cerebellar Purkinje cells, which, as a consequence, suffer from an altered trafficking, eventually culminating in a loss of cell viability, atrophy and ataxia (Liu C. et al., 2017). The above-mentioned phenotypes are the ones observed also in other neurodegenerative diseases (Huang et al., 2021). Notably, GM130 mutation leads to critical skeletal muscle developmental defects and microcephaly in zebrafish (Shamseldin et al., 2016). Collectively, these data support the idea that GM130 has an impressive role in the organisation and function of the GC. Furthermore, GM130 is crucial for the morphology, positioning and functionality of the CE during interphase, and consequently also during metaphase. Indeed, the depletion of GM130 has been observed to cause the formation of aberrant, non-functional CEs that are mislocalised above the nucleus and are deficient in nucleating the radial MT array during interphase (Kodani and Sütterlin, 2008). These aberrant interphase CEs subsequently influence the formation of non-functional multipolar spindles during mitosis, resulting in cell cycle arrest in metaphase and ultimately cell death (Kodani and Sütterlin, 2008). GM130 regulates the organisation and function of the CE by activating a Golgi-localised pool of Rho GTPase Cdc42 (Kodani et al., 2009). At the GC, GM130 forms a trimeric complex with a Golgi-localised subset of Cdc42 and a Golgi-localised subset of its specific GEF (guanine nucleotide exchange factor) Tuba. The binding of GM130 to Tuba stimulates the interaction of Tuba with Cdc42, thereby facilitating the efficient activation of Cdc42, which in turn regulates the organisation of the CEs (Kodani et al., 2009). Furthermore, an additional molecular mechanism through which GM130 controls the formation of the mitotic spindle has been elucidated (Wei et al., 2015). In the early stages of mitosis, the mitotic kinase Cdk1 phosphorylates GM130 on Ser25, which induces the dissociation of GM130 from p115. This, in turn, facilitates the mitotic disassembly of the GC into vesicles and clusters of membranes (Levine et al., 1996; Nakamura et al., 1997; Lowe et al., 1998; 2000) which concentrate around the spindle poles by metaphase (Jokitalo et al., 2001). Concurrently, Cdk1 induces nuclear envelope breakdown, thereby releasing the TPX2/importin α complex into the cytoplasm. Furthermore, Cdk1 phosphorylates importin α at Ser62, reducing its affinity for TPX2 while enhancing its interaction with GM130 (Guo et al., 2021). Consequently, GM130 binds importin α via its N-terminal classical nuclear localisation signal, thus recruiting importin α to the Golgi membranes clustered at the spindle poles and liberating the spindle assembly factor TPX2 into the cytoplasm (Wei et al., 2015). Subsequently, TPX2 interacts with AURKA which in turn triggers the nucleation of astral MTs from Golgi clusters at the spindle poles (Wei et al., 2015), thereby controlling the correct spindle orientation (Guo et al., 2021), and at the chromosomes (Kufer et al., 2002; Anderson et al., 2007). Finally, GM130 captures and bundles the nascent mitotic MTs, thereby playing a role in spindle assembly (Wei et al., 2015). It is noteworthy that GM130 has also been observed to cooperate with the MAPK (mitogen-activated protein kinase) pathway, specifically ERK3 (Li et al., 2010) and JNK2 (Huang et al., 2011), in the regulation of spindle organisation during mitosis. However, the precise molecular mechanism by which this occurs remains to be elucidated.

### 2.3 p115

p115 is a peripheral membrane protein that is localised in both the GC intermediate compartment and *cis*-Golgi vesicles. It is involved in the trafficking from the ER to the GC (Alvarez et al., 1999) and in GC reassembly after mitosis (Shorter and Warren, 1999; Dirac-Svejstrup et al., 2000). The correct functioning of p115 is regulated by phosphorylation (Brandon et al., 2003), which also mediates the interactions of p115 with GM130 and giantin (Nakamura et al., 1997; Lesa et al., 2000; Linstedt et al., 2000; Seemann et al., 2000). During the interphase, p115 interacts with GM130 via its C-terminus and γ-tubulin, a component of γTuSC and γTuRC (MT polymerisation small and large complexes), through its N-terminal armadillo fold. This interaction facilitates the recruitment of γ-tubulin to Golgi membranes, thereby enabling the formation of non-centrosomal microtubule-organising centres. Conversely, p115 localises at spindle poles throughout mitosis due to the interaction of its N-terminal armadillo-like domain with γ-tubulin, thereby playing a role in the establishment of MTOCs (centrosomal microtubule-organising centres) (Radulescu et al., 2011). Whereas the depletion of p115 leads to the complete fragmentation of GC (Puthenveedu and Linstedt, 2004; Radulescu et al., 2011), it does not affect the structure of CEs in interphase (Radulescu et al., 2011); however, it results in the loss of centrosomal integrity during mitosis. This leads to the formation of multipolar spindles with misaligned chromosomes and, ultimately, spindle collapse in late mitosis. Despite the collapse of the spindles, the p115-silenced cells do not undergo mitotic arrest or mitosis-related apoptosis. This phenotype is in contrast to that observed under the depletion of GM130 and GRASP65, where the formation of multiple CE-like structures is the result of CE overduplication in interphase, which causes aberrant spindle formation, mitotic arrest and apoptosis (Sütterlin et al., 2005; Kodani et al., 2009). These findings identify p115 as a key factor in maintaining the mitotic spindle. Furthermore, p115-silenced cells exhibit aberrant cytokinesis, characterised by the failure to form cytokinetic bridges, which lead to the generation of binucleated cells (Radulescu et al., 2011).

### 2.4 Tankyrase-1

Tankyrase-1 is a PARP (poly (ADP-ribose) polymerase) that utilises the NAD^+^ (nicotinamide adenine dinucleotide) as a substrate for the addition of multiple ADP-ribose moieties to itself and target proteins. It is a peripheral membrane protein that has been observed to localise to several subcellular structures, including the GC (Chi and Lodish, 2000; Bottone et al., 2012), spindle poles (Smith and de Lange, 1999), nuclear pore complexes (Smith and de Lange, 1999) and telomeres (Smith et al., 1998). The subcellular localisation of tankyrase-1 is subjected to change throughout the cell cycle, according to the interaction with specific binding partners. During interphase, tankyrase-1 forms a complex with GMD (GDP-Mannose-4,6-Dehydratase), the enzyme responsible for the initial step in fucose synthesis, within the cytoplasm (Bisht et al., 2012). Upon entry into mitosis the interaction between GMD and tankyrase-1 is reduced, resulting in tankyrase-1 interaction with NuMA (Nuclear Mitotic Apparatus) and TRF1. The former mediates tankyrase-1 localisation at spindle poles, while the latter is responsible for its localisation at telomeres (Bisht et al., 2012). Subsequently, in telophase, these protein interactions are lost (Smith and de Lange, 1999; Chang W. et al., 2005) causing the reassociation of tankyrase-1 with GC (Chi and Lodish, 2000). The interaction between GMD and tankyrase-1 specifically inhibits the PARP activity of tankyrase-1, thus preventing the proteasomal degradation of tankyrase-1 mediated by automodification. Similarly, this interaction has been demonstrated to inhibit the PARsylation of target proteins mediated by tankyrase-1 (Bisht et al., 2012). It is therefore proposed that the GMD-tankyrase-1 complex may serve as a readily available reservoir of tankyrase-1, maintaining the protein in an inactive state until it interacts with other binding partners. Tankyrase-1 catalytic activity is markedly elevated during mitosis and plays a central role in the correct assembly of the mitotic spindle and the maintenance of telomeric chromatin (Chang P. et al., 2005; Chang W. et al., 2005; Chang et al., 2009; Ha et al., 2012). The catalytic activity and protein interaction of tankyrase-1 are subjected to precise modulation during mitosis, facilitated by phosphorylation via a range of kinases, including GSK3 (Glycogen Synthase Kinase) (Yeh et al., 2006) and Plk1 (Ha et al., 2012). GSK3 phosphorylates tankyrase-1 on multiple serine (Ser978, Ser987 and Ser991) and threonine (Thr982) residues (Yeh et al., 2006), thereby modulating the interaction with its substrates (including NuMA) and/or other spindle-associated proteins, thus consequently promoting the efficient PARsylation and spindle formation (Sbodio and Chi, 2002; Chang P. et al., 2005; Chang W. et al., 2005; Chang et al., 2009; Yeh et al., 2006). In addition to GSK3, the mitotic serine/threonine kinase Plk1 directly binds to and phosphorylates tankyrase-1 on multiple serine and threonine residues, including Thr839, Thr930, Ser978/Thr982, and Thr1128. These phosphorylation events facilitate the localisation of tankyrase-1 at the spindle poles and telomeres, and also enhance its stability and PARP activity (Ha et al., 2012). The depletion of tankyrase-1 causes defects in bipolar spindle assembly, the formation of multipolar spindles, chromosome scattering, the lack of disjunction of sister chromatids, and MT defects such as abnormal bending angles, curling or twisting (Chang P. et al., 2005). These defects result in the activation of a Mad2-dependent spindle checkpoint, which in turn causes pre-anaphase mitotic arrest with fully paired sister chromatids (Chang P. et al., 2005). Tankyrase-1 performs these functions by modulating the structural integrity of the spindle poles and/or relevant protein interactions required for spindle structure and function through the PARsylation of several substrates (Chang P. et al., 2005). In this context, the protein targets include NuMA (Chang W. et al., 2005; Chang et al., 2009), MERIT40 (Zheng et al., 2019), TRF1 (Ha et al., 2012) and Miki (mitotic kinetics regulator) (Ozaki et al., 2012). NuMA is a coiled-coiled protein that shuttles between the interphase nuclei, the mitotic/meiotic spindle poles and the mitotic cell cortex. In these locations, it contributes to nuclear formation, bipolar spindle assembly and mitotic spindle positioning, respectively (Kiyomitsu and Boerner, 2021). It is also noteworthy that PARP3, another member of the PARP family, forms a protein complex with tankyrase-1 and NuMA. PARP3 plays a decisive role in this protein complex, whereby it markedly enhances the catalytic activity of tankyrase-1, thereby facilitating the auto-ADP ribosylation of tankyrase-1 and, subsequently, the PARsylation of NuMA. This ultimately controls specific mitotic functions, including spindle stabilisation and telomere integrity (Boehler and Dantzer, 2011; Boehler et al., 2011). In accordance with these functions, PARP3 depletion results in metaphase arrest, the accumulation of multipolar and bipolar spindles with splayed MTs, chromosome misalignment and persistent telomere fusions. These phenotypes are reminiscent of those observed following the depletion of tankyrase-1 (Chang P. et al., 2005) and NuMA (Haren et al., 2009; Silk et al., 2009). MERIT40 is a core subunit of the deubiquitinase BRISC complex, which specifically hydrolyses K63Ub (Lys63-linked polyubiquitin chains). BRISC is involved in maintaining spindle structure and function through modulating the ubiquitination level of NuMA (Yan et al., 2015). MERIT40 interacts with tankyrase-1, and this interaction is essential for the localisation of MERIT40 at spindle poles, the correct assembly of the bipolar spindle and the chromosome alignment (Zheng et al., 2019). TRF1, a duplex telomeric DNA-binding protein, is a component of a six-protein complex called shelterin, which is involved in maintaining genome stability by protecting telomeric DNA from unregulated degradation, recombination and end-to-end fusion (Smith et al., 2020). TRF1 functions as a regulator of the telomerase enzyme, controlling its access to telomeric DNA. The PARsylation of TRF1 inhibits its binding to the telomeres, thus allowing the telomerase to access the telomeres and elongate them (Muoio et al., 2022). During mitosis, the tankyrase-1-mediated PARsylation of TRF1 ensures the efficient resolution of telomeres, thus preventing the chromosome ends from undergoing telomeric fusions (Ha et al., 2012; Muoio et al., 2022). Miki, which is localised at the GC during interphase, is involved in the promotion of prometaphase. Indeed, in the late G2 to prophase transition, tankyrase-1 PARsylates Miki at the GC, which is a prerequisite for PARsylated Miki translocation to mitotic CEs and spindles, where Miki localises from the prophase to metaphase. Subsequently, during telophase Miki accumulates at the midbodies. PARsylated Miki participates to CE maturation by promoting the accumulation of γ-tubulin, GCP2, CG-NAP/AKAP450 and kendrin/pericentrin, the major components of the γ-TuRC (γ-tubulin ring complex), at mitotic CEs. Specifically, PARsylated Miki targets and anchors CG-NAP, a large scaffold protein that provides a platform for localising γ-TuRC, thus enabling the subsequent MT nucleation that is required for the proper chromosome alignment and segregation during mitosis. In accordance with this function, Miki depletion leads to prometaphase delay or arrest, chromosome misalignment and the subsequent accumulation of multinucleated cells (Ozaki et al., 2012).

### 2.5 RINT-1

RINT-1 is localised at the ER (endoplasmic reticulum), GC and CEs (Hirose et al., 2004; Arasaki et al., 2006; Lin et al., 2007). This protein is involved in a number of cellular processes through its interaction with a variety of distinct binding partners. The interaction with RAD50, a member of the SMC (structural maintenance of chromosomes) protein family, mediates RINT-1 involvement in the regulation of the G2/M checkpoint (Xiao et al., 2001). Moreover, RINT-1 acts as a scaffold protein, facilitating the interaction between p130, a member of the Rb protein family, and RAD50. This role is crucial in regulating telomere length (Kong et al., 2006). These data provide compelling evidence that RINT-1 plays a critical role in maintaining genomic stability. This role is further emphasised by the finding that RINT-1 deficiency in neuronal progenitor cells results in chromosomal aberrations including sister chromatid fusion and fusion of telomers. These chromosomal defects give rise to the formation of DNA bridges, which subsequently impair chromosome segregation (Grigaravicius et al., 2016a). RINT-1 forms a complex with ZW10 (Zeste white 10) and syntaxin 18, which modulates membrane trafficking between the ER and the GC (Arasaki et al., 2006). It is noteworthy that the interaction of ZW10 with dynein-dynactin via dynamitin, a subunit of the dynein accessory complex dynactin, mediates the movement of ZW10 along MT tracks to the centrosomal region. Here, ZW10 arrests the spindle assembly checkpoint, a surveillance mechanism that detects potential errors in the attachment of kinetochores with spindle MTs (McAinsh and Kops, 2023). It is remarkable that both dynamitin and RINT-1 bind to ZW10 in mutually exclusive manner. Furthermore, RINT-1 overexpression has been demonstrated to prevent the dynein-dynactin-mediated movement of ZW10 to the CEs (Inoue et al., 2008; Grigaravicius et al., 2016b). These findings thus indicate that RINT-1 plays a regulatory role in kinetochore attachment to the spindle through the modulation of ZW10 localisation (Inoue et al., 2008; Grigaravicius et al., 2016b). Furthermore, RINT-1 is involved in maintaining the dynamics of the GC throughout the cell cycle and CE integrity (Lin et al., 2007). Indeed, in cells where RINT-1 has been depleted, the GC loses its pericentriolar positioning and become dispersed during interphase. During mitosis, the GC is partially disassembled, resulting in an incomplete formation of Golgi haze. Finally, the GC does not reassemble around the CE during telophase (Lin et al., 2007). Furthermore, RINT-1 depletion leads to the amplification of the CE during interphase, which in turn promotes the formation of multipolar spindles and chromosome missegregation, thus leading to chromosome instability (Lin et al., 2007). The alterations in GC dynamics during cell cycle progression and the mitotic defects deriving from RINT-1 loss lead to a prolonged M phase and mitotic cell death (Lin et al., 2007). While the underlying molecular mechanisms remain to be elucidated, these findings indicate that RINT-1 is essential for the accurate coordination of GC and CE dynamics during cell division and the formation of a functional mitotic spindle.

### 2.6 Arf1 (ADP-ribosylation factor 1)

Arf1 (ADP-ribosylation factor 1) is a Ras-like GTP-binding protein that is essential for maintaining the structure and function of the GC (Donaldson and Jackson, 2000). It regulates membrane traffic at the GC and endosomes. The constitutively active Arf1 mutant has been demonstrated to impair a number of cellular processes, including ER-to-Golgi and intra-Golgi transport (Dascher and Balch, 1994), mitotic GC disassembly, chromosome segregation and cytokinesis (Altan-Bonnet et al., 2003). With regard to its involvement in mitosis, the fate and activity of Arf1 remain incompletely understood. However, there is evidence suggesting that it may play a role in mitotic GC breakdown. Arf1 is recruited to Golgi membranes by a GEF, which facilitates the conversion of Arf1-GDP into its GTP-bound form. Once activated, Arf1 recruits proteins that are necessary for DNA replication, chromosome condensation, segregation and cytokinesis, including actin, tankyrase-1, spectrin and Cdc42 (Drechsel et al., 1997; Smith and de Lange, 1999; Sütterlin et al., 2001). This process is facilitated by the binding of effector molecules (Donaldson and Klausner, 1994; De Matteis and Morrow, 2000). The inactivation of Arf1 at an early stage of mitosis could facilitate the controlled release of the aforementioned proteins into the cytoplasm, thereby ensuring the optimal progression of mitotic events. In conclusion, the model proposed by Altan-Bonnet et al. (2003) suggests that Arf1 recruitment to Golgi membranes is early inhibited during mitosis due to its conversion into an inactive GDP-bound state. This subsequently permits the detachment of peripheral proteins from Golgi membranes, leading to GC disassembly, which is a prerequisite for mitotic entry, chromosome segregation and cytokinesis. In addition, it has been speculated that, once released, the GC proteins are relocated at different subcellular structures, where they play key roles in different cellular processes including spindle formation and cytokinesis. The precise mechanism of this process is not yet fully understood, but it is undoubtedly an intriguing area for further investigation. It can be proposed that the inactivation of Arf1 represents an additional mechanism for controlling mitosis (Donaldson and Jackson, 2000).

### 2.7 STK16 (Serine/Threonine Kinase 16)

STK16 (Serine/Threonine Kinase 16) is a GC-resident enzyme that directly binds actin and regulates actin polymerisation/depolymerisation dynamics through its kinase activity. In detail, low concentrations of STK16 facilitate actin polymerisation, whereas high concentrations induce actin depolymerisation (Liu J. et al., 2017). By modulating the dynamics of actin, STK16 contributes to maintenance of GC integrity and cell cycle progression. Indeed, depletion of STK16 or inhibition of its kinase activity results in the reduction in actin filaments, indicating an alteration in actin dynamics. As a consequence, a number of effects have been observed, including the induction of GC fragmentation, the inhibition of G2/M transition and the arrest of prometaphase and cytokinesis (Liu J. et al., 2017).

## 3 Role of Golgi complex resident proteins in oogenesis

**FIGURE 4:**
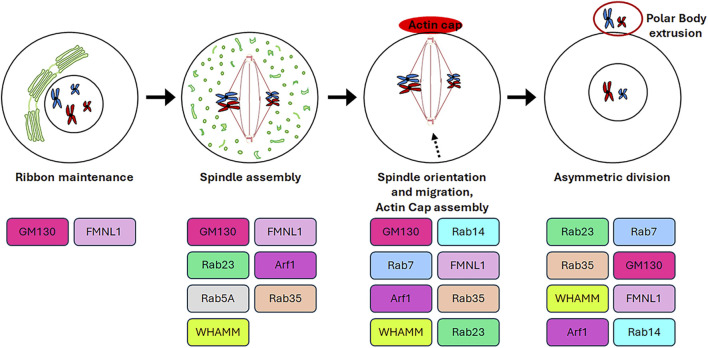
Roles of GC proteins during oogenesis. The cartoon represents schematically the roles played by the GC-resident proteins during oogenesis.

**FIGURE 5:**
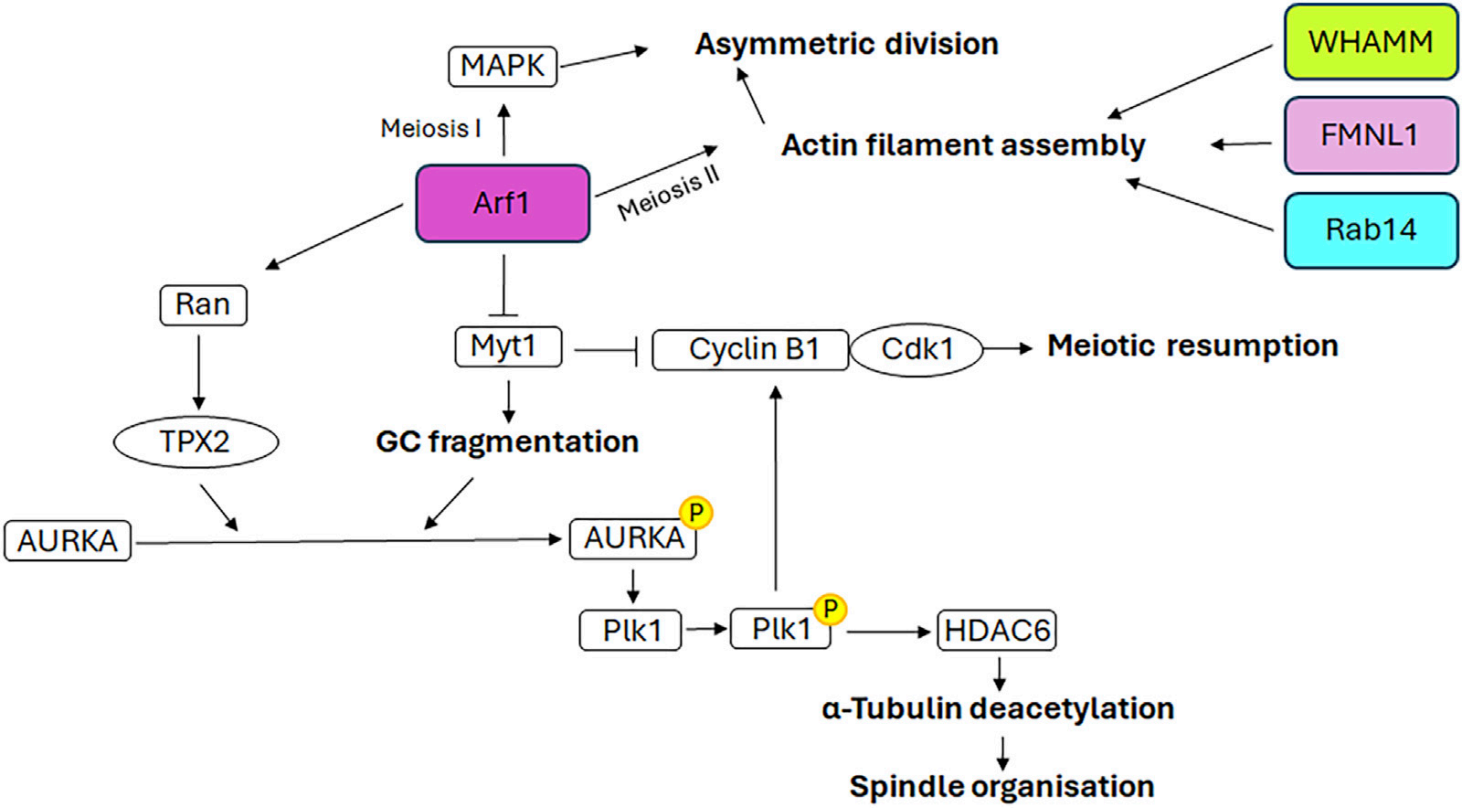
Mechanistic details of GC proteins action during oogenesis. The main pathways involving the GC-resident proteins in spindle assembly and positioning as well as meiotic resumption are displayed. Additional details on the underlying molecular mechanisms are explained in the text.

The germinal stem cells undergo an asymmetric division, resulting in the production of two daughter cells. One of these cells retains the stemness features (self-renewal), while the other undergoes a differentiation fate that culminates in the production of gametes through a distinctive cell division process known as meiosis. In contrast to mitosis, meiosis comprises two cell divisions (defined as Meiosis I and Meiosis II) and a single step of DNA replication, resulting in the generation of haploid male and female gametes (Hillers et al., 2017). In particular, in mammalian oogenesis, the two successive divisions are asymmetric and drive to the formation of two small polar bodies and the large and polarised egg, which retains all maternal components necessary for embryo development (Sun and Kim, 2013). This asymmetry is generated by the spindle orientation and migration to the cellular cortex as a result of the dynamic organisation of the cytoskeleton during both cellular divisions (Brunet and Verlhac, 2011). In contrast to mitotic cells, in which the CEs are directly responsible for the correct bipolar spindle assembly (Namgoong and Kim, 2018), mammalian oocytes lack CEs and the spindle formation is driven by multiple MTOCs (Wu and Akhmanova, 2017). Although mitosis and meiosis exhibit disparate patterns of spindle formation, they share similarities in the involvement of GC proteins in regulating the appropriate assembly of the spindle. As shown in Figure 4, the GC proteins that play a role in this process include GM130, FMNL1, Arf1, Rab proteins and WHAMM. Except for Arf1, the molecular mechanisms underlying the involvement of GC proteins in regulating spindle assembly and positioning during meiosis as well as meiotic resumption have not been fully unravelled. Therefore, Figure 5 is mainly focused on the signalling pathways through which Arf1 plays a crucial role in these processes.

### 3.1 GM130

The function of GM130 in oogenesis has been insufficiently studied and remains a topic of debate. *In vitro* experiments demonstrate that during mouse oocyte maturation GM130 regulates spindle assembly and migration, as well as asymmetric cell division (Zhang et al., 2011). The depletion of GM130 affects the localisation of proteins involved in spindle organisation, including γ-tubulin and Plk1, as well as pMEK1/2, which is involved in the extrusion of the polar body (Verlhac et al., 2000). As a consequence, the spindles are observed to be defective, exhibiting either an elongated morphology or an aberrant number of poles. Furthermore, GM130 depletion impairs spindle migration, resulting in the migration of only one of the two spindle poles to the cortex. This ultimately leads to the elongation of the spindle. If the spindle pole reaches the cortex, the extrusion of a polar body larger than that of the control oocytes is observed. Conversely, if the spindle only elongates to a minimal extent and does not reach the cortex, oocytes are arrested at metaphase I and unable to extrude the polar body. In both instances, the process of asymmetric oocyte division is disrupted (Zhang et al., 2011). Evidence suggests that GM130 performs these functions in conjunction with ERK3 (Li et al., 2010) and JNK2 (Huang et al., 2011), both of which are members of the MAPK family. However, the detailed mechanism through which this occurs has not been fully unravelled. ERK3 depletion leads to the arrest of oocyte meiosis at the metaphase I stage, a high incidence of abnormal spindles and an incorrect chromosome alignment. This is due to an interference in the attachment between kinetochores and MTs and in the activation of the spindle assembly checkpoint component BubR1 (Li et al., 2010). JNK2 co-localises with centrosomal proteins, including γ-tubulin and Plk1, and plays a role in spindle assembly and first polar body extrusion during meiosis in mouse oocytes (Huang et al., 2011). Furthermore, JNK2 has been observed at both meiotic spindle poles and the centres of cytoplasmic MT asters, thereby supporting the hypothesis that this protein may act as a component of MTOCs during meiosis (Schuh and Ellenberg, 2007).

Although these studies emphasise the role of GM130 in oocyte maturation, a more recent study performed on an oocyte-specific GM130 knockout mouse model demonstrates that the GM130 deficiency does not affect the ovulation and maturation of oocytes, and thus the murine fertility rate (Jiang et al., 2020). It is possible that these controversial findings could be explained through the *in vivo* functional redundancy of the other Golgins expressed in the mouse oocyte.

### 3.2 FMNL1 (Formin-like 1)

Formins are a family of proteins that act as actin nucleators. FMNL1 (Formin-like 1) is a member of this family. Although predominantly cytoplasmic, it also colocalises with GM130 at GC and at the spindle poles in meiotic mouse oocytes, thereby indicating its potential involvement in Golgi ribbon maintenance (Colón-Franco et al., 2011) and spindle assembly and positioning (Wang et al., 2015), respectively. FMNL1 is involved in the oocyte asymmetric division. Depletion of this protein causes defective spindle formation and the extrusion of a large polar body with a slow rate of extrusion (Wang et al., 2015). From a mechanistic perspective, FMNL1 exerts its actions through participation in multiple signalling cascades, including RhoA/FMNL1/MAPK, RhoA/FMNL1/GM130 and RhoA/FMNL1/mDia1/Profilin1. These cascades are involved in the actin assembly and spindle organisation (Fan and Sun, 2004; Xiong et al., 2008; Wang et al., 2015; Yin and Sun, 2015; Zhang et al., 2015).

### 3.3 Arf1

Arf1 has been demonstrated to participate to the mouse oocyte asymmetric division, both during meiosis I and meiosis II (Wang et al., 2009). Indeed, depletion of Arf1 causes the symmetric division during both meiotic divisions. In detail, Arf1 performs these functions through two distinct mechanisms. During meiosis I, Arf1 activates the MAPK signalling cascade, which is involved in meiosis (Fan and Sun, 2004). During meiosis II, Arf1 modulates the correct spindle orientation, which is essential for asymmetric division, through the promotion of appropriate actin filament assembly (Wang et al., 2009), in accordance with the role played in modulating the dynamics of the actin cytoskeleton (Myers and Casanova, 2008).

Recent evidence indicates that Arf1 localises to the spindle poles during murine oocyte meiosis and is a pivotal factor in meiotic resumption, MTs stability and spindle organisation (Zhang et al., 2024). Arf1 depletion induces an increase in the expression of Myt1, the inhibitory kinase of the cyclin B1/Cdk1 complex, and a decrease in the expression of cyclin B1 and Cdk1. This results in the inactivation of this complex and, consequently, the failure of meiotic resumption. Furthermore, previous studies have demonstrated the role of Myt1 in regulating the fragmentation of GC during the G2 phase and the breakdown of GC into tubules and vesicles during mitosis (Villeneuve et al., 2013). In accordance with the aforementioned evidence, the depletion of Arf1 in oocytes, through its effect on Myt1, results in an altered distribution of GC, which is no longer enriched in the spindle periphery area but is dispersed throughout the cytoplasm. Consequently, following Arf1 depletion a reduction in AURKA expression and an impairment of its activation and spindle poles localisation are observed, which in turn affect the activation of Plk1, the AURKA downstream target. This further impairs the activation of the cyclin B1/Cdk1 complex, causing the failure of meiotic resumption (Zhang et al., 2024). Moreover, the depletion of Arf1 results in additional impairment of the AURKA-Plk1 signalling pathway due to the decreased expression levels of Ran and TPX2, the downstream target of Ran and the activator of AURKA. The defective activation of AURKA-Plk1 signalling also hampers the correct assembly of spindles, which appear smaller or fragmented, by affecting the expression of the Histone deacetylase 6 and, consequently, MTs stability (Zhang et al., 2024).

### 3.4 Rab proteins

The Rab GTPase proteins are recognised for their involvement in the processes of vesicle budding, trafficking and fusion. They facilitate the movement of vesicles along the cytoskeleton through the interaction with actin and microtubule motor proteins. The relationship between Rab proteins and the cytoskeleton is of great significance, not only in the regulation of transport but also in the reorganization of the cytoskeleton (Li and Marlin, 2015). A number of Rabs have been demonstrated to influence meiotic spindle morphology and positioning, as well as the attachment of MTs to kinetochores (Shan and Sun, 2021). The following subsection presents a few illustrative examples. It should be noted that, although only Rab14 shows GC localisation, other Rabs are described here in order to emphasise the relevant role of this family of proteins in spindle formation during oogenesis.

During mouse oocyte maturation, Rab14 accumulates in the cortex and the spindle periphery. It plays a critical role in asymmetric division during meiosis. Indeed, its depletion results in defective spindle migration and positioning, which in turn causes the extrusion of large polar bodies (Zou et al., 2021). From a molecular perspective, the depletion of Rab14 induces the reduction in the expression of ROCK and phospho-cofilin, which serves as the phosphorylation target of ROCK. Furthermore, Rab14 deficiency impairs ROCK accumulation around the spindle, indicating that Rab14 modulates the RhoA/ROCK/cofilin signalling pathway (Zou et al., 2021), which mediates the actin filament assembly required for the correct spindle migration during mouse oocyte maturation (Duan et al., 2014; Duan et al., 2018). In addition to Rab14, several Rabs have been demonstrated to drive spindle organisation and migration during meiosis. Rab23 and Rab35 have been shown to control spindle organisation during oocyte meiosis by modulating tubulin acetylation (Wang et al., 2019; Zhang et al., 2019). Specifically, Rab23 accumulates at the spindle poles and promotes the migration of the motor protein Kif17 to these locations. Kif17 exerts control over MTs arrangement through its interaction with enzymes involved in the acetylation/deacetylation of tubulin, namely αTAT and Sirt2. These enzymes are responsible for the acetylation of tubulin in spindle meiotic microfilaments. The depletion of Rab23 or Kif17 leads to alterations in tubulin acetylation and, consequently, in MTs stability, which in turn perturbs spindle formation and chromosome alignment. Furthermore, the absence of Kif17 results in the reduction in cytoplasmic actin levels, which in turn affects spindle migration to the cortex. This leads to a failure in polar body extrusion and defects in mouse oocyte meiotic maturation (Wang et al., 2019). From a mechanistic perspective, Kif17 exerts control over the assembly and distribution of cytoplasmic actin through interactions with key components of the RhoA/ROCK/cofilin pathway, including RhoA, ROCK1, phospho-LIMK and phospho-cofilin, via its tail domain (Wang et al., 2019). It can therefore be concluded that the Rab23-Kif17 complex is involved in the organisation and migration of the spindle during meiosis, exerting its influence on tubulin acetylation and actin filament assembly, respectively. Similarly, Rab35 has been demonstrated to promote MTs stability and meiotic spindle formation in oocytes by modulating α-tubulin acetylation levels through its binding with Sirt2 and αTAT. Furthermore, Rab35 has been shown to interact with RhoA and control the RhoA/ROCK/cofilin pathway, thereby modulating the actin-mediated spindle migration (Zhang et al., 2019). It can be concluded that the depletion of Rab35 impairs spindle migration in oocytes as a consequence of the failure of asymmetric spindle positioning and the impairment of correct actin assembly and tubulin acetylation (Zhang et al., 2019). Similarly, Rab7 is implicated in spindle migration through its regulation of actin dynamics via interaction with actin nucleation factors. Its depletion results in aberrant spindle migration and asymmetric division defects (Pan et al., 2020). Rab5A plays a role in the establishment of the correct spindle length and kinetochore-MTs attachment during meiosis. The knockdown of Rab5A causes the formation of elongated spindles, characterised by misaligned chromosomes, due to the failure of kinetochore-MTs attachment (Ma et al., 2014). These defects are dependent on the reduction in the expression level and localisation of the nuclear matrix protein CENPF (Centromere Protein F) at kinetochores during metaphase, as well as an impairment in the disassembly of the nuclear lamina during oocyte maturation. In light of these findings, it has been proposed that the interaction between Rab5A and the nuclear lamin regulates CENPF levels and localisation at centromeres, which in turn determines the correct spindle length and kinetochore-MTs attachment. These meiotic defects can increase aneuploidy in eggs, resulting in reproductive disorders (Ma et al., 2014). While these cited proteins represent only a subset of Rab GTPase proteins involved in the proper development of meiosis, this list highlights the critical role of these proteins in meiotic spindle organisation and positioning.

### 3.5 WHAMM (WAS Protein Homolog Associated with Actin, Golgi Membranes and Microtubules)

WHAMM (WAS Protein Homolog Associated with Actin, Golgi Membranes and Microtubules) constitutes a component of the machinery responsible for the construction and maintenance of the actin cytoskeleton. Indeed, it is a nucleation-promoting factor which activates the Arp2/3 complex (actin-related protein 2/3 complex) (Rottner et al., 2010). It is localised in the *cis*-Golgi and tubulovesicular ERGIC (ER-Golgi intermediate compartment), where it interacts with both the actin and MT cytoskeletons, thereby regulating the membrane tubulation and dynamics during transport from the ER to the GC (Campellone et al., 2008). WHAMM is expressed during all stages of oocyte maturation and localises at the meiotic spindle actin, a structure constituted by actin filaments which permeate the spindle and are involved in spindle formation, maintenance and migration, thus controlling chromosome alignment (Mogessie and Schuh, 2017; Plessner et al., 2019). During oocyte maturation, WHAMM is involved in the formation and migration of the spindle to the cortex of the oocyte (Huang et al., 2013; Jo et al., 2021). Indeed, the depletion of WHAMM impairs the formation of spindle actin and the MTOC clustering and migration at the spindle poles. This results in the formation of aberrant bipolar spindles, which are characterised by increased spindle length and chromosome misalignment. This, in turn, has been demonstrated to drive increased chromosomal aneuploidy (Jo et al., 2021). Furthermore, WHAMM depletion affects the correct actin pattern in the oocyte and causes the disruption of actin cap formation, thus impairing spindle migration (Longo and Chen, 1985; Leader et al., 2002). This, in turn, causes the extrusion of a large polar body and a failure of asymmetric division (Huang et al., 2013).

## 4 Golgi complex as a storage of signalling molecules regulating asymmetric cell division and cell fate

**FIGURE 6:**
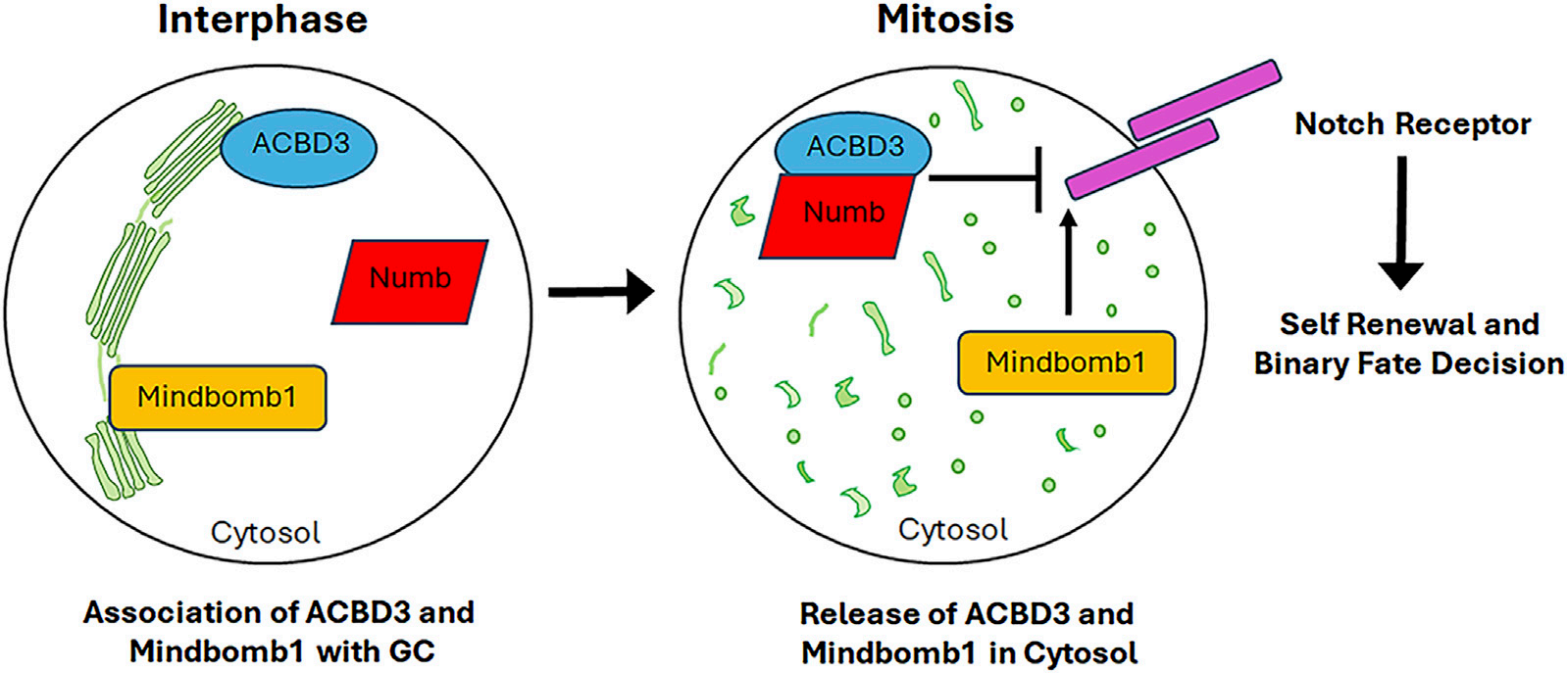
GC serves as storage centre for signalling molecules involved in cell fate commitment. Upon mitotic GC fragmentation, proteins associated to the GC and involved in the commitment of cell fate are released into the cytosol thus affecting the cell fate decision. See the text for further details.

The accumulating evidence indicates that the GC plays a role in asymmetric cell division, whereby both stem and progenitor cells generate a daughter stem and progenitor cell (self-renewal), respectively, and a cell with a differentiation committed fate. The generation of daughter cells with intrinsic differences requires the initial polarisation of the mother cell, which determines the formation of two distinct cellular sides. Subsequently, cell fate determinants (including proteins, organelles and even RNA) are localised to only one side, and then the mitotic spindle is aligned along the axis of cell polarity. This results in the segregation of cell fate determinants predominantly into one of the two daughter cells (Sunchu and Cabernard, 2020; Chao et al., 2024). In the mammalian central nervous system, the Notch signalling pathway is responsible for controlling binary fate decisions and plays a significant role in the maintenance of progenitor cells (Pierfelice et al., 2011). Indeed, Notch regulators, including Numb and Mindbomb1, have been demonstrated to function as fate determinants. Numb is the primary determinant of cell fate. Numb inhibits Notch signalling by recruiting components of the ubiquitination machinery to the Notch receptor, thereby promoting ubiquitination of the Notch at the plasma membrane and subsequent degradation of the Notch intracellular domain (McGill and McGlade, 2003). The function of Numb in determining cell fate has been well documented in the context of the embryonic nervous system. The asymmetric segregation of cytosolic Numb to only one of the two daughter cells results in an asymmetric cell division, thereby specifying the progenitor fate over the neuronal fate (Petersen et al., 2002; Petersen et al., 2004). However, Numb is also expressed in the neuronal daughter cell (Zhong et al., 1997), where it is also essential for neuronal survival and differentiation (Huang et al., 2005). The dual function of Numb in sustaining the progenitor cell and promoting neuronal differentiation can be attributed to the GC-mediated subcellular distribution of the Numb interactor ACBD3 (Acyl-CoA binding domain containing 3). ACBD3 interacts with Numb and this interaction is necessary for the determination of cell fate. ACBD3 localises at the GC in neurons and interphase progenitor cells. The fragmentation of the GC during mitosis releases ACBD3 into the cytosol, where it is free to bind Numb and thus specify the fate of the progenitor cell (Figure 6). During telophase, ACBD3 reassociates with the reforming GC. It can thus be concluded that the process of GC fragmentation and reconstitution during the cell cycle regulates the subcellular distribution of ACBD3, which in turn affects the activity of Numb. The latter is only able to specify the progenitor fate during mitosis and/or shortly afterwards, when its partner ACBD3 is also present in the cytosol. Subsequently, following cell division, the association of ACBD3 with the GC enables newly synthesised Numb proteins to promote neuronal differentiation through their involvement in a distinct signalling pathway (Zhou et al., 2007). Another Notch pathway regulator involved in neural stem cells asymmetric division is Mindbomb1, a mono-ubiquitin ligase that modulates the Notch ligands trafficking and promotes their activity (Weinmaster and Fischer, 2011). In asymmetric cell division, Mindbomb1 co-localises asymmetrically with centriolar satellite proteins PCM1 and AZI1 at the daughter centriole in interphase (Tozer et al., 2017). The asymmetric localisation of Mindbomb1 results in the generation of daughter cells with differing levels of this protein. Subsequently, the daughter cell with the highest concentration of Mindbomb1 undergoes differentiation. Mindbomb1 regulates fate decisions through the unequal activation of Notch in daughter cells (Tozer et al., 2017). Furthermore, an additional pool of Mindbomb1 was identified to be associated with the GC during interphase. Upon entering mitosis, the Golgi-associated pool of Mindbomb1 is released into the cytosol due to GC fragmentation (Figure 6) and subsequently recruited to the mother CE, thereby compensating for the centrosomal asymmetry of Mindbomb1. The symmetric localisation of Mindbomb1 at both spindle poles drives to the equal inheritance of Mindbomb1 by the daughter cells, which in turn induces reciprocal Notch activation between daugther cells. This process ultimately leads to the symmetric proliferative division, which generates two progenitor cells (Tozer et al., 2017). In summary, the GC serves as a repository for both cell fate determinants, such as Mindbomb1, and their interactors, such as ACBD3. Its fragmentation during mitosis enables the release of these factors, thereby linking cell fate specification with cell cycle progression.

## 5 Involvement of cell division-related GC-resident proteins in cancer

As previously outlined, GC plays a pivotal role in the regulation of cell division, encompassing both mitosis and meiosis. When the structure and/or function of several GC-resident proteins that regulate these processes is dysregulated, cells may undergo chromosomal instability and/or altered proliferation, which are two of the hallmarks of cancer (Hanahan and Weinberg, 2011; Donizy and Marczuk, 2019). Indeed, an increasing body of evidence indicates that the mutation of genes encoding GC proteins involved in cell division may be a potential trigger for cancer onset (Liu et al., 2021). To gain further insight into this topic, we initially gathered data on the genetic alterations of these GC proteins in cancer from the publicly available TCGA database (https://www.cbioportal.org/). While the 16 proteins under discussion in this review have been shown to undergo genetic alterations in several tumour types, for the sake of brevity Table 1 presents only the tumour type in which the highest rate of genetic alteration has been observed for each of them. The data presented in Table 1 were gathered without patient stratification (i.e., without considering stage, gender, race, grade, mutation burden, and neoantigen load) and show the rate of genetic alterations (source https://www.cbioportal.org), the correlation between overall survival and gene expression, and the number of patients for each data set (source https://www.kmplot.com, Pan-cancer RNA-seq). In addition, Table 2 lists the genetic mutations detected in the tumour with the highest rate of genetic alteration for each GC-resident protein. For further details about the genetic alterations, the correlation with overall survival and the genetic mutations in the other cancer types, the reader is directed to the following sources: https://www.cbioportal.org and https://www.kmplot.com. It is noteworthy that the tumour in which 8 out of the 16 GC proteins examined are predominantly mutated is uterine corpus endometrial carcinoma (source: TCGA database, PanCanAtlas), which is one of the most prevalent gynecological malignancies (Zhao and Li, 2023). It is of note that 2 (GRASP65 and Rab5A) of the remaining 8 proteins exhibit high mutation rates in the same cancer type, although these rates are not the highest (see Table 1 and the website https://www.cbioportal.org). Furthermore, the correlation between the gene expression of each GC-localised protein and overall survival has been analysed in the tumour in which these proteins exhibit the highest rate of genetic alteration. The Kaplan-Meier Plotter indicates that elevated expression of GM130, p115 and STK16 is associated with improved overall survival, whereas high expression of ACBD3 is correlated with reduced overall survival. No correlation was identified between gene expression and overall survival for the remaining proteins (Table 1). This information is in accordance with the findings of previous studies which have demonstrated that these GC proteins could play a role in the development of a number of tumour types. The behaviour of GM130 in cancer is controversial, with studies indicating that it is either downregulated [e.g., in colorectal and breast cancers (Baschieri et al., 2014)] or upregulated [e.g., in gastric cancer, (Zhao et al., 2015); in lung cancer (Chang et al., 2012)] in certain cancer types. The inconsistency of the role of GM130 in affecting tumourigenesis suggests that it plays different functions in a tumour context-dependent manner, thus underlying the necessity to further deepen this aspect. Tankyrase-1 is a well-established contributor to cancer development (Seimiya, 2006; Haikarainen et al., 2014; Sagathia et al., 2023), particularly in the context of colorectal cancer (Masuda et al., 2015; Thorvaldsen, 2017). p115 has been demonstrated to stimulate cancer in multiple organs. Indeed, p115 has been identified as a mutated gene in different cancers, including liver cancer (Yoon et al., 2021), gastric cancer (Li et al., 2013), colon cancer (Sui et al., 2015), myeloma cells (Jin and Dai, 2016) and B-cell acute lymphoblastic leukemia (Jaiswal et al., 2021). STK16 is a pivotal player in regulating GC structure. Its overexpression has been linked to the development of triple negative breast cancer (Zhu et al., 2024) and the promotion of cancer progression in lung (Wang et al., 2022) and colon (Peng et al., 2024) tissues. WHAMM functions as a tumour suppressor in leukemia (Biber et al., 2021) and is an unfavourable prostate cancer marker, whereas it is a favourable cervical cancer marker (https://www.proteinatlas.org/ENSG00000156232-WHAMM/pathology). FMNL1 is an oncogene with a high mutation rate in numerous cancer models (Zhang et al., 2020). Indeed, in some cases, it is downregulated, for example in glioblastoma (Higa et al., 2019), although it is often overexpressed, which has an unfavourable impact on the prognosis (Nie et al., 2020). Examples of such studies include those on gastric cancer (Mansuri et al., 2021), leukemia (Favaro et al., 2013; Chen et al., 2018), lymphomas (Favaro et al., 2006; Gardberg et al., 2014) and kidney cancer (Ma et al., 2021). The function of RINT-1 in tumour formation remains a topic of debate, as it has been observed to act as both a tumour suppressor and an oncogene. Indeed, RINT-1 haploinsufficiency in Rint-1^+/−^ mice promotes chromosomal instability, which in turn stimulates the development of multiple tumours including lymphomas, lung cancers, hepatocellular carcinomas, uterus and breast tumours. These findings suggest a role for RINT-1 as a tumour suppressor (Lin et al., 2007). Conversely, alternative evidence suggests that RINT-1 acts as an oncogene. The Rint-1 genomic locus is amplified in multiple tumour types, including glioblastomas (Beroukhim et al., 2010; Quayle et al., 2012) and is overexpressed and mutated in human colorectal cancers (Otterpohl and Gould, 2017). Furthermore, it has been demonstrated to stimulate *in vivo* tumour formation when over-expressed in primary non-transformed astrocytes (Quayle et al., 2012). RINT-1 is frequently referenced as a breast cancer, colon cancer and Lynch syndrome predisposition gene (Park et al., 2014). However, an alternative study has not provided any evidence to support the role of RINT1 as a cancer susceptibility gene, thus emphasising the necessity for further investigation (Li et al., 2016). The preferential amplification of Mindbomb1 in pancreatic cancer with a worse prognosis has been demonstrated to promote metastatic progression and chemoresistance (Fu et al., 2020). ACBD3 is typically upregulated and plays a role in promoting tumour formation, particularly in breast invasive carcinoma (Huang et al., 2018) and gastric cancer (Zheng et al., 2021). In both cases, increased ACBD3 expression is associated with poor survival outcomes. The role of GRASP65 in cancer remains a topic of debate, with evidence suggesting that decreased expression may contribute to carcinogenesis (Bui et al., 2021; Bajaj et al., 2022). Arf1 overexpression has been demonstrated to stimulate the proliferation and invasion of prostate, breast, and ovarian cancer cells (Boulay et al., 2011; Davis et al., 2016; Schlienger et al., 2016; Gu et al., 2017). Furthermore, elevated Arf1 expression in human breast cancer specimens is associated with a poor prognosis for the patient (Schlienger et al., 2016). The aberrant expression of Rab proteins has been documented in numerous cancer types (Cheng et al., 2005; Chia and Tang, 2009). Rab14 is overexpressed in gastric, ovarian and bladder cancers and functions as an oncogene, stimulating tumour proliferation and aggressiveness (Hou et al., 2016; Guo et al., 2017; Deng et al., 2023). Similarly, Rab35, which is involved in regulating cell division, has been demonstrated to act as an inducer of tumourigenesis (Gibieža and Petrikaitė, 2021). Rab23 is overexpressed in diffuse-type gastric cancer, where it serves as an invasion mediator gene (Hou et al., 2008), in hepatocellular carcinoma, where its overexpression correlates significantly with tumour size (Liu et al., 2007), and in muscle-invasive FGFR3-non-mutated bladder cancers, suggesting its active role in tumour progression (Ho et al., 2012). The role of Rab7A in carcinogenesis is debated (Guerra and Bucci, 2019). Indeed, it has been demonstrated to serve as a tumour promoter in breast cancer and cholangiocarcinoma (Suwandittakul et al., 2017; Xie et al., 2019), while it acts as a tumour suppressor in prostate cancer and glioblastoma (Steffan et al., 2014; Wang et al., 2017). Furthermore, elevated levels of expression have been observed in human gastric cancer specimens, with increased expression correlating with a poor clinical prognosis (Liu et al., 2020). Rab5A is overexpressed in several cancer types, including breast and ovarian cancers, hepatocellular carcinoma and oral squamous cell carcinoma (Zhao et al., 2010; Yang et al., 2011; Pan et al., 2015; Geng et al., 2016; Zhang et al., 2017; Ma et al., 2020), where it acts as an oncoprotein, stimulating cancer proliferation and invasion and thus promoting the malignant phenotype.

**TABLE 1:** Cell division-related GC-resident proteins in cancer.

Gene	Tumour type showing the highest rate of genetic alterations^a^	Rate of genetic alterations (%)^b^	Correlation between gene expression and overall survival^c^	Number of patients in each data set without patient stratification^c^
GM130	Uterine Corpus Endometrial Carcinoma	7.19	High expression high survival (logrank *p* = 0.052)	n = 543
Tankyrase-1	Uterine Corpus Endometrial Carcinoma	10.97	The expression does not correlate with overall survival	n = 543
p115	Uterine Corpus Endometrial Carcinoma	4.92	High expression high survival (logrank *p* = 0.024)	n = 543
STK16	Uterine Corpus Endometrial Carcinoma	4.35	High expression high survival (logrank *p* = 0.00053)	n = 543
WHAMM	Uterine Corpus Endometrial Carcinoma	4.92	The expression does not correlate with overall survival	n = 543
FMNL1	Uterine Corpus Endometrial Carcinoma	7.57	The expression does not correlate with overall survival	n = 543
Rab14	Uterine Corpus Endometrial Carcinoma	2.65	The expression does not correlate with overall survival	n = 543
Rab35	Uterine Corpus Endometrial Carcinoma	2.27	The expression does not correlate with overall survival	n = 543
RINT-1	Esophageal Adenocarcinoma	8.24	The expression does not correlate with overall survival	n = 80
Mindbomb1	Pancreatic Adenocarcinoma	14.13	The expression does not correlate with overall survival	n = 177
ACBD3	Breast Invasive Carcinoma	9.22	High expression low survival (logrank *p* = 0.013)	n = 1,090
Arf1	Breast Invasive Carcinoma	9.14	The expression does not correlate with overall survival	n = 1,090
Rab7A	Lung Squamous Cell Carcinoma	6.78	The expression does not correlate with overall survival	n = 501
Rab5A	Bladder Urothelial Carcinoma	4.14	The expression does not correlate with overall survival	n = 405
Rab23	Diffuse Large B-Cell Lymphoma	4.17	Not available	Not available
GRASP65	Skin Cutaneous Melanoma	3.6	Not available	Not available

^a^ The source of data is the website TCGA, PanCancer Atlas at link https://www.cbioportal.org.

^b^ The genetic alterations include mutations, amplifications, duplications, deletions, multiple alterations and structural variants. The source of data is the website TCGA, PanCancer Atlas at link https://www.cbioportal.org.

^c^ The source of data is the website Kaplan-Meier Plotter Pan Cancer RNA-seq at the link https://www.kmplot.com.

**TABLE 2:** Genetic mutations of the cell division-related GC-resident proteins detected in the tumor type showing the highest rate of genetic alterations.

Gene	Tumour type showing the highest rate of genetic alterations^a^	Genetic mutations (detected in the tumour type showing the highest rate of genetic alterations)^b^						
		Frameshift insertion	Frameshift deletion	Inframe deletion	Fusion	Missense	Nonsense	Splice
GM130	Uterine Corpus Endometrial Carcinoma	None detected	E516Sfs*42 Y989Tfs*9	None detected	None detected	A569T R896C R537C A480T R521C A397V A800T E313D V647M R811H L321F Y846F S869N H106N Q961R R473Q M528I E750G S953Y A659V R532H R674C A520V E343D V432A L86I E586K R703Q N110S T70I R896H E884V A358V R543Q R976C R257G G917W A511T	E887*	X382_splice X118_splice
Tankyrase-1	Uterine Corpus Endometrial Carcinoma	L562Pfs*10	T508Hfs*33 S1221Afs*34 H1100Tfs*59	S134del	None detected	R383Q A1271T R1076H R646H R79L R1200Q R1044W R1180H G367E R1149Q L951V T833I T938M V1284I A209T P405L H467Y A549V G693C V300M N1071D A1202V R781I D709N D432Y A806T G1092D V635L A430V D794Y L1305I A244T L841M E441G D501A A443T G1233C A950S E479K A691T M574I	R1149* E419* E959* E1064* Q1166*	X300_splice X344_splice X527_splice X1,149_splice X557_splice X423_splice
p115	Uterine Corpus Endometrial Carcinoma	N227Kfs*10	N227Tfs*18	None detected	None detected	S533L T110N R168C G524V K627E L305I L906M L915P L392R Q242R A161V G238D R243H T599M S754F A780T T638I L94F T285A F234V A426T L688F T885A	Q669* R327* E85*	X171_splice X211_splice
STK16	Uterine Corpus Endometrial Carcinoma	None detected	None detected	None detected	MAFF-STK16 Fusion	R50Q P162L R79H W127R D166E R70C R79C D213N S180F K142R Q304H R19C L52P R215W R280C F230L G157E	R64*	X219_splice
WHAMM	Uterine Corpus Endometrial Carcinoma	None detected	None detected	P658del	None detected	A737T A731V R677Q R698H S558C S503N R773C S799G C435R R549C I747M R420I F621C G270D A248V E346D K608N D265Y M313I P578T L339F L255F V307A L760F A353V S792Y L482V	E502* E280* E283* R345*	X262_splice X312_splice
FMNL1	Uterine Corpus Endometrial Carcinoma	None detected	A81Qfs*7 V521Cfs*99	None detected	None detected	A1018T G243D R920H R875H V919M S950L V229I A707V R731H G858D A907V K979N G1063V E976D V1071A R1080H R992H R122W E759D V221I R641Q E256D K264N A155S N341H R219H F986I E889D K633N F990C Q75H E311D E166G P1038S A1064T R860W	E413*	X44_splice X134_splice X999_splice X298_splice
Rab14	Uterine Corpus Endometrial Carcinoma	F36Ifs*4	K35Nfs*18 T157Rfs*5	None detected	None detected	T67M I44L E137G A168V Y8C R110M L121I G45S R51I	None detected	X157_splice
Rab35	Uterine Corpus Endometrial Carcinoma	None detected	None detected	None detected	None detected	A139T D89N S22N E94K K173E V157M I47L N109D	None detected	None detected
RINT-1	Esophageal Adenocarcinoma	None detected	None detected	None detected	None detected	R710W S295P R157C	None detected	X729_splice
Mindbomb1	Pancreatic Adenocarcinoma	None detected	None detected	None detected	MIB1-GREB1L Fusion	S841P D198N	None detected	X926_splice
ACBD3	Breast Invasive Carcinoma	None detected	E348Nfs*21	None detected	None detected	E226K R523T E348Q E212Q	None detected	None detected
Arf1	Breast Invasive Carcinoma	None detected	E17Kfs*27	None detected	None detected	R149H A137T	None detected	None detected
Rab7A	Lung Squamous Cell Carcinoma	None detected	None detected	None detected	None detected	A135S S101I	None detected	None detected
Rab5A	Bladder Urothelial Carcinoma	None detected	None detected	None detected	None detected	Y151S S107F	None detected	None detected
Rab23	Diffuse Large B-Cell Lymphoma	None detected	None detected	None detected	None detected	None detected	None detected	None detected
GRASP65	Skin Cutaneous Melanoma	None detected	None detected	None detected	None detected	N62S P203Q L316F E358D Q435K P207H P27T P298Q R106S P305Q G197W G357C	E344*	None detected

^a^ The genetic alterations include mutations, amplifications, duplications, deletions, multiple alterations and structural variants. The source of data is the website TCGA, PanCancer Atlas at link https://www.cbioportal.org.

^b^ The genetic mutations include frameshift insertions and deletions, inframe deletions, fusions, missense, nonsense and splicing variants. The data source is the website TCGA, PanCancer Atlas at link https://www.cbioportal.org.

The collective evidence thus suggests that these GC proteins play a fundamental role in the biology of cancer cells. The functions of the aforementioned proteins may appear to be contradictory in certain instances. However, it is probable that these proteins act in a manner that is dependent on the specific cell and tumour context in which they bind different interactors, which may influence their activity. Consequently, further studies are required in order to specifically address the role of these GC-resident proteins in the process of tumourigenesis.

## 6 Discussion and conclusion

The GC plays a pivotal role in cellular homeostasis. Indeed, its structural and functional dysregulation has been linked to the development of several diseases, including cancer, diabetes, inflammation, infectious illnesses, neurodegenerative and cardiovascular diseases (Zappa et al., 2018; Liu et al., 2021; Lee et al., 2024). The underlying molecular mechanisms of these disorders are primarily associated with alterations in vesicular trafficking and glycosylation. In the context of cancer, alterations in GC morphology lead to decreased levels of key GC components such as giantin, GRASP65 and GM130. As a consequence, multiple cellular processes are impacted, including protein glycosylation, vesicular trafficking, extracellular matrix remodeling and acidification, autophagy and redox homeostasis. This ultimately facilitates cancer cell proliferation and malignant progression (Lee et al., 2024). Furthermore, several GC-localised proteins (such as GM130, Arf1, p115, PAQR3, GOLM1 and Golgin97) have been demonstrated to regulate signalling cascades involved in carcinogenesis including the MAPK, Wnt, TGFβ, NF-κB and PI3K/AKT pathways (Spano and Colanzi, 2022). In addition to the aforementioned molecular mechanisms, evidence suggests that several GC-resident proteins are involved in cell cycle progression through regulating the proper centrosome maturation, spindle assembly and chromosome segregation. Consequently, structural and functional abnormalities affecting GC-resident proteins may result in aberrant cell division, ultimately leading to aneuploidy and cancer. This review elucidates the roles played by the GC-localised proteins in promoting the maturation of the CE/MTOC as well as the formation and positioning of the spindle during mitosis and meiosis and highlights the molecular mechanisms underlying these actions. It is noteworthy that some of the proteins examined in this review have been identified as players in the process of tumour formation, functioning as either tumour suppressors or oncogenes. It is possible that some of these proteins may appear to have a controversial role; however, this is likely due to the influence of the cell and tumour context on their behaviour, which is dependent on the interaction with specific interactors. The relevance of GC-resident proteins in the pathogenesis of cancer identifies them as potential biomarkers for both diagnosis and prognosis, as well as potential therapeutic targets for treatment. Among the GC-resident proteins discussed in this review, WHAMM is a prostate cancer marker with unfavourable prognostic implications, whereas it is a favourable cervical cancer marker (https://www.proteinatlas.org/ENSG00000156232-WHAMM/pathology). The overexpression of Arf1 has been identified as a predictor of poor clinical outcome in patients affected by triple negative breast cancer (Schlienger et al., 2016). FMNL1 has been identified as an independent predictor of poor prognosis and a promising therapeutic target in glioblastoma multiforme, clear cell renal cell carcinoma and gastric cancer (Higa et al., 2019; Nie et al., 2020; Ma et al., 2021). Similarly, increased ACBD3 expression serves as a prognostic biomarker of poor survival in breast (Huang et al., 2018) and gastric (Zheng et al., 2021) cancers. Moreover, given that ACBD3 targeting in cellular and mouse models of breast cancer impairs tumourigenesis, it may represent a suitable therapeutic target for the breast cancer treatment (Huang et al., 2018). Experiments performed on lung cancer cellular and mouse models have demonstrated the therapeutic efficacy of GM130 targeting in lung cancer treatment (Chang et al., 2012). Moreover, the multiple functions played by tankyrase-1 suggest that this enzyme represents a promising molecular target for cancer therapy (Lakshmi et al., 2017; Thorvaldsen, 2017). It is noteworthy that, in addition to the aforementioned proteins, additional GC proteins have been identified as potential cancer biomarkers. For instance, TMEM165 (transmembrane protein 165) and serum GP73 (Golgi glycoprotein 73) have been identified as potential biomarkers for hepatocellular carcinoma diagnosis (Liu et al., 2011; Morota et al., 2011; Lee et al., 2018). On the same line of evidence, elevated nuclear expression of GS28 is associated with unfavourable outcomes for patients diagnosed with cervical and colorectal cancers (Cho et al., 2016; Lee et al., 2017).

Over time, the research has led to the development of a wide variety of drugs that target the GC through multiple mechanisms, including the targeting of glycosylation, Golgi trafficking, proteins or oncogenes involved in cancer development and progression as well as the targeting of the STING (stimulator of interferon genes) pathway, an innate immune response highly dependent on vesicular trafficking (Zappa et al., 2018; Liu et al., 2021; Martins et al., 2023; Vlad et al., 2023; Lee et al., 2024). Nevertheless, despite the demonstration of anti-cancer activity of several molecules on cellular and animal models of diverse cancer types, no pharmacological agent targeting specifically the GC has been approved for clinical use in cancer treatment (Zappa et al., 2018; Liu et al., 2021; Martins et al., 2023; Vlad et al., 2023; Lee et al., 2024). This evidence underscores the necessity for further investigation into the molecular mechanisms through which GC proteins exert their activity. The findings could lead to the identification of novel, more sensitive and specific biomarkers for diagnosis and/or prognosis, as well as molecular targets for cancer therapy. In addition, given that candidate therapeutic targets have already been identified, future research should aim to design and develop small molecules for pharmacological intervention with high targeting specificity and limited side effects, with a view to exploring novel therapeutic avenues.

## Abbreviations

GC, Golgi complex; CE, centrosome; MT, microtubule; GRASPs, Golgi Reassembly And Stacking Proteins; AURKA, Aurora-A; MTOCs, microtubule-organising centres; Plk3, Polo-like kinase 3; Plk1, Polo-like kinase 1; Cdk1, cyclin-dependent kinase 1; JNK, c-Jun N-terminal kinase; MAPK, mitogen-activated protein kinase; PARP, poly (ADP-ribose) polymerase; GMD, GDP-Mannose-4,6-Dehydratase; NuMA, nuclear mitotic apparatus; GSK3, Glycogen Synthase Kinase; Miki, mitotic kinetics regulator; ER, endoplasmic reticulum; ZW10, Zeste white 10; Arf1, ADP-ribosylation factor 1; STK16, Serine/threonine kinase 16; ACBD3, Acyl-CoA binding domain containing 3; TCGA, The Cancer Genome Atlas.
